# Prevalence and factors associated with second trimester pregnancy loss among women admitted at a National Referral Hospital in Uganda: a cross-sectional study

**DOI:** 10.4314/ahs.v24i4.26

**Published:** 2024-12

**Authors:** Charles Irumba, Dan K Kaye, Justus K Barageine, Rodgers Ampwera, Hope Atwiine

**Affiliations:** 1 Department of obstetrics and gynaecology, School of Medicine, College of Health Sciences, Makerere University; 2 Department of Internal Medicine, Kiruddu National Referral Hospital

**Keywords:** Second trimester pregnancy loss or abortion, prevalence, associated factors

## Abstract

**Background:**

Second trimester pregnancy loss (abortion) refers to induced or spontaneous termination of pregnancy from 13 to the end of 26 weeks of gestation. Second trimester abortions contribute to a high proportion of maternal morbidity, mortality and psychological stress especially in low-resource countries with restricted access to safe abortion services. While globally, the prevalence of second trimester abortions is 10–15%, the prevalence at Kawempe National Referral Hospital was not known. The study objective was to determine the prevalence and factors associated with second trimester abortion at Kawempe National Referral Hospital.

**Methods:**

A hospital based cross-sectional study was conducted among 235 women with abortions admitted at Kawempe National Referral Hospital. Quantitative data was collected using a structured interviewer administered questionnaire. Bivariate and multivariate analysis was done using STATA version 14. The Student's T- test was used to check for association between the dependent and independent variables.

**Results:**

The prevalence of second trimester abortion was 41.7%.|Factors associated with second trimester abortion were; being HIV positive (aPR = 1.9, 95% CI =1.32 – 2.61), having a urinary tract infection (aPR = 1.9, 95% CI =1.30 – 2.72), malaria infection confirmed by a rapid test (aPR = 2.5, 95% CI= 1.75 – 3.54), no prior history of abortion (aPR = 1.5, 95% CI =1.04 – 2.27) and no condom use in the previous 1 year (aPR = 1.6, 95% CI =1.11 – 2.23).

**Conclusion:**

The prevalence of second trimester abortion was higher than the global estimates. Service providers should actively screen pregnant women for HIV, urinary tract infection, malaria and then offer effective treatment accordingly. The Ministry of Health should develop and disseminate protocols and standard management guidelines for case management of second trimester abortions.

## Background

Second trimester pregnancy loss (abortion) refers to spontaneous or induced termination of pregnancy from 13 to the end of 26 weeks of gestation. Twenty six weeks is the age of viability in Uganda^1^. This comprises 10-15% of approximately 42 million abortions that occur annually worldwide 2, 3. These second trimester pregnancy losses are associated with more morbidity, mortality, social and psychological challenges as compared with first trimester abortions^2^. The lack of necessary skills among healthcare providers and the absence of dedicated protocols and guidelines for managing second trimester abortions contribute to this problem 4,6,7.

In sub-Saharan Africa, 2/3 of all abortion complications are attributable to the second trimester period of which most of them are unsafe abortions 9. According to Guttmacher Institute and Ipas, more than one-third of all women with abortion complications were seeking care after second trimester abortion and it was more common among women who lived in rural areas than among their urban counterparts in Ethiopia 10.

In Ethiopia, the prevalence of second trimester abortion in 2015 was 33.6% 2 and about 33% in 2020 Other studies in South Africa showed a prevalence of second trimester abortion to be 20% 12, and in Vietnam 8-11%^13^. Research on this topic in other settings showed that the factors associated with second trimester abortion and its complications were, low use of modern contraceptive methods 9, 10, repressive abortion laws and policies, gender discrimination and lack of safe abortion services, stigma associated with abortion, family or partner pressure 14, some patients with spontaneous second trimester abortions had no apparent causes.

Uganda is among the low income countries in Sub-Saharan Africa with poor reproductive health indicators especially a high Maternal Mortality Ratio of 336 per 100000 live births^5^. Uganda has restrictive abortion laws in the penal code act, whereby abortion can only be medically induced if the pregnancy puts the mother's life in jeopardy or the fetus has confirmed congenital malformations that are incompatible with extra-uterine life. These laws are not associated with lower abortion rates. This coupled with negative moral and social judgment increase the rates of unsafe pregnancy terminations and even those that have spontaneous abortions have stigma and delay to get professional medical assistance. Poorly managed abortion and its complications are among the top five causes of maternal mortality in Uganda contributing about 7% of all maternal deaths 6. Second Trimester pregnancy losses or abortions cause a significantly higher morbidity and mortality as compared to first trimester abortions 8.

The prevalence of second trimester abortion and its associated factors at Kawempe National Referral Hospital was unknown, hindering the implementation of measures to reduce complications, particularly maternal morbidity and mortality^15^. The study's findings could fill that evidence gap, enhance the management of second trimester abortions, reduce the burden of the associated complications, inform the Ugandan Ministry of Health policy, guide the development of protocols and procedures, and inspire further research on this topic. 2

## Methods

### Study design and setting

This was a hospital based cross-sectional study conducted in October and November of 2020 in the Emergency Gynaecology ward at Kawempe National Referral Hospital. It is the largest National Referral Hospital for Maternal and Child health services in Uganda with a bed capacity of 170, located in Kawempe division of Kampala City. It also serves as a teaching hospital for the Department of Obstetrics and Gynecology, School of Medicine, College of Health Sciences, Makerere University.

The Hospital has a relatively busy emergency gynaecology ward with an attendance of about 30-40 patients daily, with about 10-15 having abortions and related complications. For abortions, mothers areadmitted for surgical or medical management immediately upon contact with the doctor on duty. We enrolled into the study all women with any type of abortion 0 to 26 weeks of gestation that were admitted during the study period, were above 18 years and emancipated minors that provided informed consent. We excluded women with confirmed gestational trophoblastic disease and those with threatening abortion. The gestation age was determined from the first day of the last normal menstrual period or by ultra sound scan done in early pregnancy prior to the abortion

### Sample size and sampling technique

Using the Leslie Kish formula^16^, a sample size of 200 participants was obtained using the prevalence of Second trimester abortion of 15.3% obtained in Zambia 17. When weused Fleiss formula for sample size cdetermination, a total of 235 participants was obtained using results from a study conducted in Ethiopia 2. This bigger sample size was considered for this study. The participants were consecutively recruited into the study until the required sample size was attained.

### Data collection and quality assurance

Data collection was done in the months of October and November 2020, using interviewer-administered, structured, pre-tested, Luganda language-translated questionnaires by the principal investigator and 5 midwives as research assistants. They were trained by the principal investigator for five days on the objectives, relevance of the study, informed consent process, how to conduct the interviews and how to perform rapid diagnostic tests while ensuring confidentiality and all the ethical issues involved. Additional information was obtained from the participant's treatment records. Participants were interviewed after receiving the first four of the five components of postabortion care on the ward 18. The questionnaires were made anonymous and assigned unique codes to maintain privacy. We obtained permission from the institutional review board to seek informed consent from emancipated minors for the study. We ensured privacy for all participants, including emancipated minors, during questionnaire administration. Each morning, the principal investigator reviewed all questionnaires for completeness, consistency, and accuracy of information collected. The questionnaires were securely stored, with limited access granted only to the principal investigator and the five research assistants.

### Data analysis

There was double data entry into Epi data version 4.2, it was cleaned and exported to STATA version 14 for analysis. Continuous variables were summarized as means and standard deviation for normally distributed data, as well as medians and interquartile ranges for skewed data. Categorical variables were summarized using frequencies and percentages. The prevalence of second trimester abortion was calculated using the numerator as the number of cases with second trimester abortion and the denominator as total number of all abortion cases admitted in the emergency gynaecology ward during the study period. Bivariate analysis was used to check for associations between the dependent and independent variables. Categorical variables were summarized as proportions and analyzed by Chis-square tests. Student's T- test was used to check for association between continuous variables and the dependent variable. For multivariate analysis, variables which had a p-value < 0.2 at bivariate analysis, logistic regression was used. Confounding was assessed using multiple logistic regression. The strength of association was assessed using prevalence ratios. The 95% confidence interval was used and a p-value of ≤ 0.05 was considered statistically significant.

### Ethical considerations

Permission to conduct the study was obtained from the Department of Obstetrics and Gynecology, Kawempe National Referral Hospital management and approval was obtained from the School of Medicine Research and Ethics Committee of Makerere University #REC REF 2020-162. Written informed consent was obtained from adult participants, emancipated minors, and from next of kin for other minors. Assent was obtained from other minors prior to their recruitment into the study.

## Results

### Socio-demographic characteristics of the study participants

There were 235 participants recruited into the study. Age ranged from 15-45 years, with with age category 20-24 years being the majority. The median age was 25 years (interquartile range (IQR) was 22-30 years). The details and other characteristics are indicated in table 1.

**Table 1:** Socio-demographic characteristics of the study participants

Variable	Frequency(n=235)		Percentage (%)
**Age (median, IQR)**		25 (22, 30)	
**Age of respondents (years)**			
15 - 19	35		14.9
20 - 24	93		39.6
25 - 29	57		24.2
>30	50		21.3
**Marital status**			
Single	67		28.5
Married	168		71.5
**Parity (Number of pregnancies)**			
One	69		29.4
Two	51		21.7
Three	45		19.1
Four	30		12.8
Greater or equal to five	40		17.0
**Education level of respondent**			
None	7		3.0
Primary	76		32.3
Secondary	132		56.2
Tertiary	20		8.5
**Employment status**			
Not employed	84		35.7
Employed	133		56.6
Student	18		7.7
**Religion of respondent**			
Catholic	61		26.0
Anglican	67		28.5
Moslem	51		21.7
Other*	56		23.8
**Residence of respondent**			
Urban	20**1**		85.9
Rural	33		14.1
**Monthly Income (UGX)**			
<100,000	128		54.5
100000 – 200000	57		24.3
>200,000 - 300000	17		7.2
>300,000	33		14.0

Note; *Pentecost, Seventh Day Adventist, UGX; Ugandan Shillings

### Abortion-related characteristics of the study participants

Table 2 shows that 54.9% of the participants had intended pregnancies and over 70% did not have a prior history of abortion. About 82.9% of the previous abortions were spontaneous. Over 57% of the participants had one previous abortion. The participants with previous second trimester abortions were 37.1%.

**Table 2:** Abortion related characteristics of the study participants

Variable	Frequency (n=235)	Percentage (%)
**Intended pregnancy**		
Yes	129	54.9
No	106	45.1
**History of abortion**		
Yes	70	29.8
No	165	70.2
**Type of previous abortion**		
None	165	
Induced	12	17.1
Spontaneous	58	82.9
**Number of previous abortions**		
None	165	
Once	40	57.1
Twice	22	31.4
Thrice or more	8	11.5
**Trimester of previous abortion**		
None	165	
First trimester	44	62.9
Second trimester	26	37.1
**Had an incompetent cervix**		
Yes	18	7.7
No	217	92.3
**Fetal congenital anomalies**		
Yes	4	1.7
No	231	98.3
**Current form of abortion**		
Induced	34	14.5
Spontaneous	201	85.5
**Intervention used to terminate pregnancy**		
None	201	
Modern drugs	23	67.7
Traditional medicine	5	14.7
Instrumentation	6	17.6

### Prevalence of second trimester pregnancy loss (abortion)

During the study period, 98 of the 235 participantshad second trimester pregnancy loss (abortions) and therefore the prevalence was 41.7%.

### Factors associated with second trimester abortion

These were; individuals who had never had an abortion before were 1.5 times more likely to experience a second trimester abortion compared to those who had had a prior abortion (aPR = 1.5, 95% CI =1.04 - 2.27), individuals who had not used condoms in the previous one year were 1.6 times more likely to experience a second trimester abortion compared to those who had used the condoms (aPR = 1.6, 95% CI =1.11 – 2.23), individuals that were HIV positive were 1.9 times more likely to have second trimester abortions compared to those that were HIV negative (aPR = 1.9, 95% CI =1.32 – 2.61), participants that hada urinary tract infection were 1.9 times more likely to have second trimester abortions that those that did not have a urinary tract infection (aPR = 1.9, 95% CI =1.30 – 2.72) and lastly participants that had confirmed malaria were 2.5 times more likely to have second trimester abortions than those that did not have malaria(aPR = 2.5, 95% CI 1.75 – 3.54). The details are indicated in table 3.

**Table 3:** The bivariate and multivariate logistic regression results on factors associated with second trimester abortion

Variable	Category		Unadjusted PR (95% CI)	P value	Adjusted PR (95% CI)	P value

	First trimester Frequency (%)	Second trimester Frequency (%)				
**Current form of abortion**						
Spontaneous	121 (60.2)	80 (39.8)	reference		reference	
Induced	16 (47.1)	18 (52.9)	1.3 (0.93 - 1.91)	0.121	1.3 (0.90 – 1.83)	0.169
**History of abortion**						
Yes	49 (70.0)	21 (30.0)	reference		reference	
Non	88 (53.3)	77 (46.7)	1.6 (1.05 - 2.31)	0.028	1.5 (1.04 - 2.27)	0.030*
**Ever used contraceptives**						
Yes	91 (62.3)	55 (37.7)	reference		reference	
No	46 (51.7)	43 (48.3)	1.3 (0.95 - 1.73)	0.104	1.3 (0.94 - 1.73)	0.114
**Use of condoms in last 1 year**						
Yes	49 (65.3)	26 (34.7)	reference		reference	
No	88 (55.4)	71 (44.6)	1.3 (0.90 - 1.84)	0.164	1.6 (1.11 - 2.23)	0.012*
**HIV status**						
Negative	129 (60.9)	83 (39.1)	reference		reference	
Positive	8 (34.8)	15 (65.2)	1.7 (1.18 - 2.35)	0.004	1.9 (1.32 - 2.61)	<0.001*
**Urinary Tract Infection**						
No	125 (60.4)	82 (39.6)	reference		reference	
Yes	12 (42.9)	16 (57.1)	1.4 (1.01 - 2.07)	0.048	1.9 (1.30 - 2.72)	0.001*
**Malaria**						
Negative	134 (60.4)	88 (39.4)	reference		reference	
Positive	3 (23.1)	10 (76.9)	1.9 (1.38 - 2.73)	<0.001	2.5 (1.75 - 3.54)	<0.001*

Note; *statistical significance at p-value <0.05

## Discussion

The prevalence of second trimester pregnancy loss (abortion) during the study period was 41.7% (98/235) which is higher than the global average of 10-15% 17.

his prevalence was still higher than the 33.6% found in Ethiopia in 2015 2 and then about 33% in 2020 11.One potential reason for the higher prevalence might be attributed to the fact that our study took place in a national referral hospital, serving a broader geographic area, and therefore receiving patients from across the country who have complications that lower-level facilities couldn't handle. Additionally, existing literature highlights that second trimester abortions tend to have more severe complications compared to those in the first trimester. Moreover, differences in socio-demographic characteristics among the study populations could have also contributed. Another factor worth considering is the occurrence of clandestine induced abortions, particularly given Uganda's restrictive abortion laws and the negative moral and social judgments from the communities where these participants resided.

Participants that had no history of abortion were 1.5 times more likely to have a second trimester abortion compared to those that had a prior history of abortion. This finding differed from that of a study done at Debre Markos Referral Hospital in Ethiopia where they found an association between having a history of abortion and a subsequent second trimester abortion 11. There was no plausible scientific explanation for this finding in our study. Women whose first pregnancy resulted in second trimester abortion were at a higher risk of having the second pregnancy also resulting in second trimester abortion compared with women who had a live birth 19. This could be explained by having recurrent urinary tract infection, incompetent cervix, and other recurrent causes. Participants that did not use condoms in the previous one year were 1.6 times more likely to have a second trimester abortion compared with their counterparts who used condoms regularly. This could be explained by the fact that, those that do not use condoms regularly easily get unwanted pregnancies and sexually transmitted infections which are all individually associated with second trimester abortions 11. HIV positive participants were almost two times likely to have second trimester abortion than those that were HIV negative. This finding was consistent with several studies that have proven the association between HIV infection and second trimester abortion. The possible explanation is the systemic inflammation which also affects the placental bed 20. Having a urinary tract infection (UTI) conferred almost twice the risk of having a second trimester abortion compared to those without. This finding was consistent with several studies that have proven the association between recurrent UTI and second trimester abortion. Having a UTI can lead to the release of pro-inflammatory substances, such as cytokines, that can affect the uterine environment and potentially lead spontaneous pregnancy loss^21^,^20^,^22^. Having a confirmed diagnosis of malaria in pregnancy using a rapid diagnostic test kit led to a 2.5 times risk of developing second trimester abortion compared with those that did not have malaria. This finding was in agreement with the findings of a study conducted in Thailand 23. The association between malaria and abortion was also confirmed in a Ugandan prospective cohort study with intensive malaria screening and prompt treatment but also in other studies from different study settings^24^. There is sequestration of Plasmodium falciparum- infected red blood cells in the intervillous spaces of the placenta causing parasitaetemia. There is release of inflammatory cytokines (IFN-γ, IL-2 and TNF-α) at the placental bed and cervix which cause placenta insufficiency through microvascular damage, ischaemia and micro-infarcts in the placenta, cervical ripening and hence second trimester abortions.^23^

## Conclusion

The prevalence of second trimester abortion among women with abortions admitted at Kawempe National Referral Hospital was higher than the global average. The factors associated with second trimester abortion were; being HIV positive, having a urinary tract infection, having malaria confirmed on a rapid diagnostic kit, no prior history of abortion and no condom use in the previous 1 year. Therefore, antenatal care providers should actively scren women for HIV, urinary tract infection, malaria and then offer effective treatment accordingly. The Ministry of health in Uganda should quickly develop and disseminate a separate comprehensive protocol and standard operating procedures for case management to clinicians. A qualitative study is needed to expound on the factors associated in order to guide evidence-based and patient-centered clinical practice

## Study limitations

Some clients lacked precise information about their Last Normal Menstrual Period (LNMP) and didn't undergo an ultrasound scan before abortion, making it challenging to objectively classify them as having a second trimester abortion. This could have influenced the reported prevalence of second trimester abortions. Social desirability bias might have led to inaccurate responses, although efforts were made to ensure privacy and confidentiality. Additionally, recall bias could have affected responses regarding past events. The study did not have a qualitative component to investigate the outcomes and coping strategies of participants after a second trimester pregnancy loss, which could have provided a more comprehensive understanding of the topic.

## Data Availability

The datasets used and/or analysed during the current study are available from the corresponding author on reasonable request.
